# Tired Nature’s Sweet Restorer

**Published:** 1886-06

**Authors:** 


**Tired Nature's Sweet Restorer.**—One of the most economical medicines that mortals can use is sleep. It is a sovereign remedy for weakness; it cures restlessness, uneasiness and irritability; it will remedy headache; it also cures nervousness. When weary we should rest; when exhausted we should sleep. To resort to stimulants is suicidal; what weary men need is sleep. The lack of sleep causes neuralgia, paralysis and insanity. Many a person dies for want of sleep, and the point where many a sufferer turns his feet from the very gates of death to the open path of life is when he sinks to sleep. Of almost every sick man it may be said, as of Lazarus, "If he sleeps he will do well." Another excellent medicine is sunshine. The world requires more of it, morally and physically. It is more soothing than morphine, more potent than poppies. It is good for liver complaint, for neuralgia, for rheumatism, for melancholy—for everything. Make your rooms sunny and cheerful; build your houses so as to command the sunshine all day long.

